# Association of vitamin D deficiency with post-stroke depression: a retrospective cohort study from the TriNetX US collaborative networks

**DOI:** 10.3389/fnut.2023.1236233

**Published:** 2023-08-04

**Authors:** Chun-Ning Ho, Cheuk-Kwan Sun, Jheng-Yan Wu, Jen-Yin Chen, Ying-Jen Chang, I-Wen Chen, Kuo-Chuan Hung

**Affiliations:** ^1^Department of Anesthesiology, Chi Mei Medical Center, Tainan City, Taiwan; ^2^Department of Hospital and Health Care Administration, College of Recreation and Health Management, Chia Nan University of Pharmacy and Science, Tainan City, Taiwan; ^3^Department of Biotechnology and Food Technology, Southern Taiwan University of Science and Technology, Tainan City, Taiwan; ^4^Department of Emergency Medicine, E-Da Dachang Hospital, I-Shou University, Kaohsiung City, Taiwan; ^5^School of Medicine for International Students, College of Medicine, I-Shou University, Kaohsiung City, Taiwan; ^6^Department of Nutrition, Chi Mei Medical Center, Tainan City, Taiwan; ^7^College of Medicine, National Sun Yat-sen University, School of Medicine, Kaohsiung City, Taiwan; ^8^Department of Anesthesiology, Chi Mei Medical Center, Liouying, Tainan City, Taiwan

**Keywords:** post-stroke depression, vitamin D deficiency, propensity score matching, stroke, vitamin D

## Abstract

**Background:**

Post-stroke depression (PSD) affects up to one-third of patients who survive stroke. This matched cohort study aimed to investigate the relationship between vitamin D deficiency (VDD) and PSD using a global health research network.

**Methods:**

Adult patients with first-ever stroke were eligible for inclusion if their circulating vitamin D levels were available within 3 months before the onset of stroke. Patients were subdivided into those with VDD [VDD group, 25(OH) D < 20 ng/mL] and those with normal vitamin D levels [control group, 25(OH) D: 30–80 ng/mL]. By using propensity score matching (PSM), potential confounding factors were adjusted. The primary outcomes were the association of VDD with the risk of PSD at the 3-month and 12-month follow-ups, while the secondary outcomes were the relationships between VDD and the risk of pneumonia as well as emergency department visits at the 12-month follow-up.

**Results:**

After PSM, 758 individuals were included in each group, with no significant differences in baseline characteristics. Musculoskeletal diseases, metabolic disorders, and hypertension were the three leading comorbidities in both the groups. The incidence of PSD was not significantly different between the two groups at the 3-month (5.8% vs. 4.7%, *p* = 0.358) and 12-month (11.6% vs. 10.2%, *p* = 0.364) follow-up. VDD was not associated with an increased risk of PSD at the 3-month [hazard ratio (HR) = 1.258, *p* = 0.358] or 12-month follow-up (HR = 1.210, *p* = 0.364). In addition, VDD was not associated with an increased risk of pneumonia (HR = 1.053, *p* = 0.823) or emergency visits at the 12-month follow-up (HR = 1.206, *p* = 0.148).

**Conclusion:**

The results revealed no significant link between VDD and PSD risk during the 3-month and 12-month follow-up periods, suggesting that VDD might not play a substantial role in PSD risk. However, further extensive studies employing a prospective design are necessary to explore the potential protective effects of vitamin D against PSD and validate these findings.

## 1. Introduction

Post-stroke depression (PSD), a common psychiatric disorder that usually occurs in the first few months after stroke, affects up to one-third of patients who survive stroke. The incidence may vary depending on the population, diagnostic criteria, and choice of assessment tool (1). In Asian stroke survivors, PSD reportedly affects approximately 26.6 to 29.1% (2, 3). A cohort study of 157,243 stroke patients revealed that 25.4% of them developed depression within two years (4). Although the exact causes of PSD are not fully understood, they may involve various factors such as dysregulation of the hypothalamic–pituitary–adrenal (HPA) axis, elevated expression of inflammatory markers, reduced levels of monoamines, excitotoxicity mediated by glutamate, and abnormal neurotrophic responses (5–8). Patients with PSD are at a higher risk of short– and long-term mortality (9, 10). Additionally, PSD may be associated with a higher probability of cognitive impairment (11), an elevated risk of suicide (12), increased hospitalization costs (13), a greater risk of falls (14), and a lower quality of life than stroke survivors without PSD (15–17). Advanced age, female sex, history of depression, left hemispheric stroke, more severe disease, and genetic predisposition (e.g., apolipoprotein E) have been linked to the development of PSD (1). In 2020, stroke emerged as the primary cause of both mortality and disability worldwide, with a total of 15.5 million cases reported globally (18). Given the substantial number of stroke patients and the notable prevalence of PSD among stroke survivors, it is imperative to identify the relevant risk factors and implement preventive strategies for PSD.

Vitamin D is a group of fat-soluble secosteroids primarily obtained through dermal synthesis after exposure to Ultraviolet-B radiation (19). The well-known biological action of vitamin D is the promotion of enterocyte differentiation and intestinal calcium absorption, thereby facilitating calcium homeostasis (19). In addition to enhancing skeletal growth and repair, vitamin D is known to play important physiological roles in the regulation of the immune system, maintenance of healthy muscle function, and suppression of inflammation (19–21). Several studies have reported an association between vitamin D deficiency (VDD) and depressive symptoms in adults aged over 65 years and women in the postpartum period (22–25), suggesting a link between low levels of vitamin D and depression. The underlying mechanisms between VDD and the development of depression remain unknown; however, they may be associated with three main mechanisms: regulating serotonin production, reducing inflammation, and acting as an antioxidant to counter oxidative stress (26–29).

Several small-scale studies focusing on Asian populations have also suggested a correlation between low levels of vitamin D and PSD (2, 3, 30). Nevertheless, the limited number of patients included in these studies (2, 3, 30) and the inclusion of only Asians may limit the extrapolation of their findings. To address these issues, this matched cohort study aimed to investigate the relationship between VDD and PSD using a global health research network (i.e., TriNetX).

## 2. Methods

### 2.1. Study design, data source, and ethical statement

This was a retrospective observational study based on the TriNetX Diamond research network (Cambridge, MA, United States) that provides access to the electronic medical records (diagnoses, procedures, medications, laboratory values, and genomic information) of approximately 108 million patients across 76 healthcare organizations. Data for this study were gathered on February 20, 2023.

TriNetX is a global collaboration platform that houses de-identified healthcare data. Only de-identified patient records were collected and no individually identifiable information was used, collected, or transmitted. The organization strictly follows the Health Insurance Portability and Accountability Act (HIPAA) and maintains an Information Security Management System (ISMS) to ensure the protection of healthcare data it access. Accordingly, TriNetX has received a waiver from the Western IRB owing to the de-identification of patient data in accordance with Section x 164.514(a) of the HIPAA Privacy Rule. Additionally, the use of TriNetX in the current study was approved by the Institutional Review Board of the Chi Mei Medical Center (IRB Serial No. 11203–010).

### 2.2. Patient population

Patients aged >20 years with first-ever stroke were potentially eligible for inclusion if their circulating vitamin D levels (i.e., 25-Hydroxyvitamin D3 and 25-Hydroxyvitamin D2 level) were available within three months before the onset of stroke. The index date was defined as the first time a stroke was diagnosed. Patients were subdivided into those with VDD (i.e., the VDD group) and those with normal vitamin D levels (i.e., the control group). In both groups, patients were excluded for (1) a previous history of stroke, vitamin D insufficiency, dementia, Parkinson’s disease, dysphasia and aphasia, osteoporosis, or major depression, or (2) expiring within one year. Additionally, patients in the control group were excluded if they had a diagnosis of VDD within one year prior to the onset of stroke.

The ICD-10-CM (International Classification of Diseases, Tenth Revision, Clinical Modification) codes for disease diagnoses are as follows: non-traumatic subarachnoid hemorrhage [ICD10: I60]; non-traumatic intracerebral hemorrhage [ICD10: I61]; other and unspecified non-traumatic intracranial hemorrhage [ICD10: I62]; cerebral infarction [ICD10: I63]; sequelae of cerebrovascular disease [ICD10: I69]; non-pyogenic thrombosis of the intracranial venous system [ICD10: I67.6]; cerebral ischemia [ICD10: I67.82]; cerebral autosomal dominant arteriopathy with subcortical infarcts and leukoencephalopathy [ICD10: I67.850]; depressive episode [ICD10: F32]; major depressive disorder, recurrent [ICD10: F33]; vascular dementia [ICD10: F01]; dementia in other diseases classified elsewhere [ICD10: F02]; unspecified dementia [ICD10: F03]; Parkinson’s disease [ICD10: G20]; secondary Parkinsonism [ICD10: G21]; dysphasia and aphasia [ICD10: R47.0]; osteoporosis with current pathological fracture [ICD10: M80]; and osteoporosis without current pathological fracture [ICD10: M81].

### 2.3. Data collection

Baseline data on age, sex, race, and comorbidities, including hypertension, diabetes mellitus, ischemic heart diseases, metabolic disorders, disorders of the thyroid gland, musculoskeletal diseases, pain, nonpsychotic mental disorders, insomnia, mood disorders, non-mood psychotic disorders, and psychoactive substance use, were collected.

### 2.4. Outcomes and definitions

The primary outcome of this study was the association of VDD with the risk of PSD at the 3-month and 12-month follow-ups. VDD was defined as a 25(OH) D level < 20 ng/mL. Patients with serum 25(OH)D between 20–30 ng/mL were classified as having vitamin D insufficiency. On the other hand, patients with serum 25(OH)D between 30–80 ng/mL were considered to have normal vitamin D levels. Regarding the secondary outcomes, we investigated the relationships between VDD and the risk of pneumonia, as well as emergency department visits at the 12-month follow-up. These secondary outcomes were selected based on previous research reporting an association between VDD and an increased risk of community-acquired pneumonia or emergency department visits (31, 32). Accordingly, we aimed to investigate the potential impact of VDD on these outcomes in stroke survivors.

### 2.5. Propensity score matching and statistical analysis

Prior to data analysis, we adjusted for confounding factors by integrating propensity score matching (PSM) into the analysis section of the TrinetX platform. Logistic regression was adopted to generate propensity scores and then applying “greedy nearest neighbor matching” with a caliper of 0.1 pooled standard deviations. Our cohorts were matched based on age at the index date, sex, race, and comorbidities. Absolute values of >0.1 standardized mean difference on covariate balance were considered positive for covariate imbalance. Statistical significance was defined as a two-sided alpha value <0.05. Hazard ratios (HR) with 95% confidence interval (95% CI) were calculated for all outcomes. Sensitivity analyses based on the use of psychoactive substances (i.e., yes vs. no) or stroke subtype (i.e., hemorrhagic stroke vs. ischemic stroke) were performed to examine the consistency of the primary outcomes at the 12-month follow-up. The TriNetX user interface was used for risk analysis and Kaplan–Meier survival analysis. Differences between the two groups in Kaplan–Meier survival analysis were calculated using a log-rank test based on a Cox proportional hazard model. Kaplan–Meier graphical curves were drawn from the output statistics downloaded from TrinetX using the pandas, matplotlib, and seaborn packages in Spyder, a Python development environment.

## 3. Results

### 3.1. Patient selection and matching

**Figure 1:**
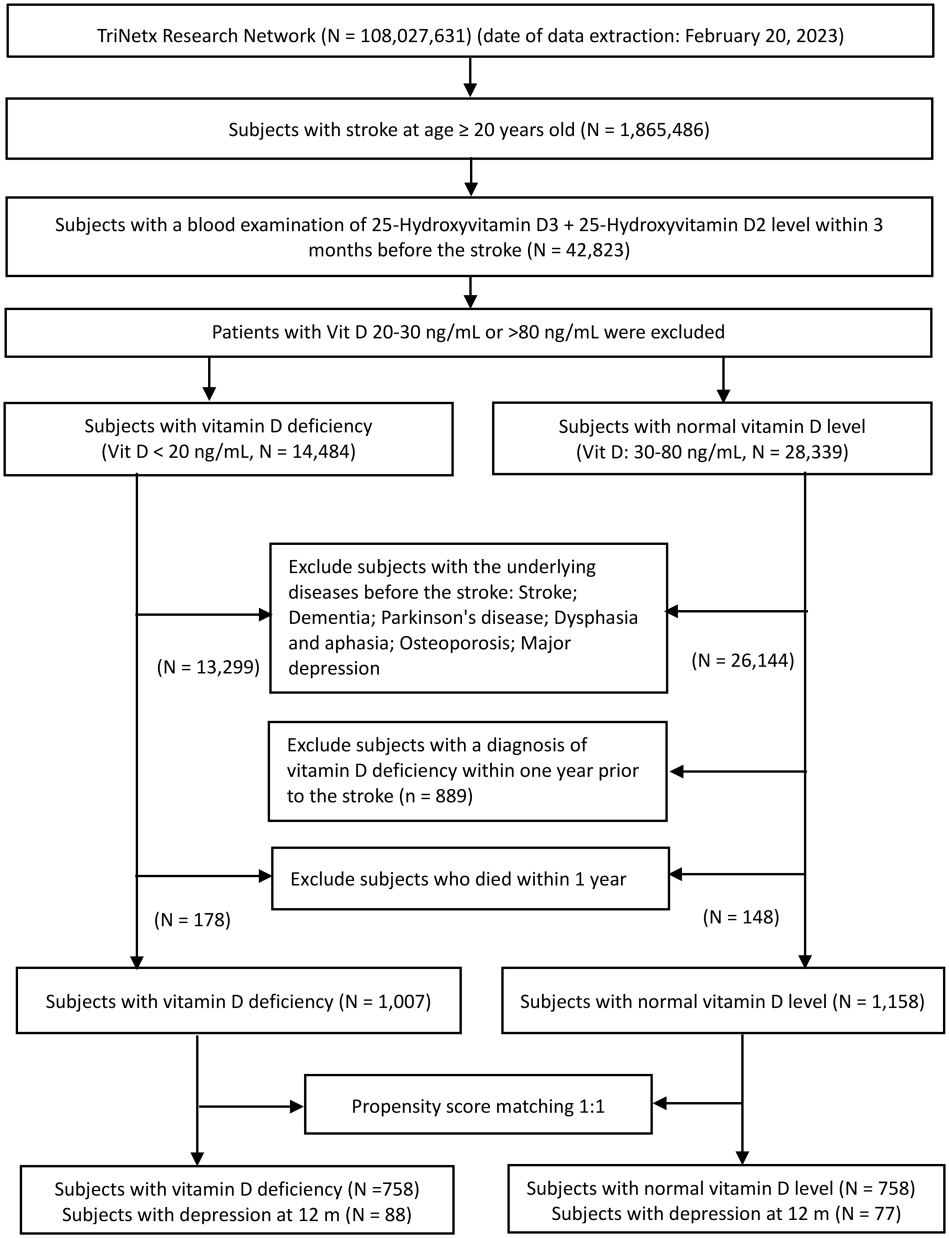
A flow chart showing the selection of patients.

A flowchart of patient selection is shown in Figure 1. Of the 108,027,631 adults identified on February 20, 2023, in TrinetX’s research network, 1,865,486 adult patients with first-ever stroke were considered eligible after screening. Following the exclusion of patients without information on circulating vitamin D levels, data from 42,823 individuals were retrieved for analysis. Based on circulating vitamin D concentrations within three months prior to the index stroke event, eligible patients were divided into two groups. While 14,484 subjects had a vitamin D level ≤ 20 ng/mL (VDD group), 28,339 had a vitamin D level between 30 and 80 ng/mL (control group). After excluding patients with a history of stroke, dementia, Parkinson’s disease, aphasia, dysphasia, osteoporosis, and depression prior to the date of index stroke and those who died within one year after the index stroke date, 1,007 patients met the eligibility criteria for inclusion in the VDD cohort. Potentially eligible subjects in the control group (i.e., vitamin D level 30–80 ng/mL) were excluded if they were diagnosed with VDD within one year of the index event. Finally, 1,158 subjects with normal vitamin D levels met the criteria for the control group. After 1:1 propensity score matching, 758 individuals were included in each group (Figure 1).

### 3.2. Characteristics of patients at baseline and after matching

Before PSM, the age at the index date was lower in the VDD group than in the control group (56.2 ± 15.4 vs. 62.0 ± 14.9, respectively, *p* < 0.001). The proportion of females was slightly lower in the VDD group than in the control group (53.6% vs. 59.9%, respectively; *p* = 0.003). Both groups had Caucasian dominance, followed by African American dominance (Table 1). The proportion of Caucasians was lower in the VDD group than in the control group (53.7% vs. 76.4%, *p* < 0.001), while the proportion of African Americans was higher in the former than in the latter (30.7% vs. 12.5%, *p* < 0.001).

**Table 1:** Baseline characteristics of study subjects (before and after Propensity score matching).

Variables	Before matching				After matching			
	VDD group (*N* = 1,007)	Control group (*N* = 1,158)	*p*- value	SMD	VDD group (*N* = 758)	Control group (*N* = 758)	*p*- value	SMD
Age at index date								
Mean ± SD	56.2 ± 15.4	62.0 ± 14.9	< 0.001	0.380	58.9 ± 14.6	58.5 ± 15.3	0.612	0.026
Gender								
Female	540 (53.6%)	694 (59.9%)	0.003	0.128	419 (55.3%)	424 (55.9%)	0.796	0.013
Male	467 (46.4%)	464 (40.1%)	0.003	0.128	339 (44.7%)	334 (44.1%)	0.796	0.013
Race								
White	541 (53.7%)	885 (76.4%)	< 0.001	0.490	509 (67.2%)	507 (66.9%)	0.913	0.006
Black or African American	309 (30.7%)	145 (12.5%)	< 0.001	0.453	138 (18.2%)	141 (18.6%)	0.842	0.010
Asian	17 (1.7%)	26 (2.3%)	0.354	0.040	16 (2.1%)	18 (2.4%)	0.729	0.018
Unknown Race	132 (13.1%)	95 (8.2%)	< 0.001	0.159	89 (11.7%)	85 (11.2%)	0.747	0.017
Underlying disease								
Hypertensive Diseases	542 (53.8%)	557 (48.1%)	0.008	0.115	390 (51.5%)	368 (48.6%)	0.258	0.058
Diabetes Mellitus	371 (36.8%)	304 (26.3%)	< 0.001	0.229	244 (32.2%)	231 (30.5%)	0.472	0.037
Ischemic Heart Diseases	196 (19.5%)	203 (17.5%)	0.247	0.050	147 (19.4%)	144 (19.0%)	0.845	0.010
Metabolic Disorders	545 (54.1%)	568 (49.1%)	0.019	0.102	396 (52.2%)	375 (49.5%)	0.281	0.055
Disorders of Thyroid Gland	208 (20.7%)	290 (25.0%)	0.016	0.105	167 (22.0%)	167 (22.0%)	1	0
Neoplasms	288 (28.6%)	411 (35.5%)	< 0.001	0.148	233 (30.7%)	238 (31.4%)	0.781	0.014
Diseases of the Musculoskeletal System	606 (60.2%)	773 (66.8%)	0.002	0.137	473 (62.4%)	449 (59.2%)	0.207	0.065
Pain, unspecified	256 (25.4%)	332 (28.7%)	0.090	0.073	204 (26.9%)	200 (26.4%)	0.816	0.012
Nonpsychotic Mental Disorders	208 (20.7%)	271 (23.4%)	0.125	0.066	160 (21.1%)	162 (21.4%)	0.900	0.006
Insomnia	52 (5.2%)	62 (5.4%)	0.843	0.009	43 (5.7%)	38 (5.0%)	0.568	0.029
Mood Disorders	28 (2.8%)	27 (2.3%)	0.508	0.028	20 (2.6%)	19 (2.5%)	0.871	0.008
Non-mood Psychotic Disorders	24 (2.4%)	20 (1.7%)	0.280	0.046	15 (2.0%)	13 (1.7%)	0.703	0.020
Psychoactive Substance Use	240 (23.8%)	210 (18.1%)	0.001	0.140	165 (21.8%)	162 (21.4%)	0.851	0.010

SMD, Standard mean difference; VDD, vitamin D deficiency.

In contrast to the control group, the VDD group had a higher prevalence of hypertensive disease (53.8% vs. 48.1%, *p* = 0.009), diabetes mellitus (36.8% vs. 26.3%, *p* < 0.001), metabolic disorders (54.1% vs. 49.1%, *p* = 0.019), and psychoactive substance use (24.8% vs. 18.2%, *p* < 0.001). However, a larger number of patients with neoplasms and musculoskeletal diseases were included in the control group. After 1:1 matching, there was no difference in baseline characteristics between the two groups. Musculoskeletal diseases, metabolic disorders, and hypertension were the three leading comorbidities in both the groups.

### 3.3. Cumulative risk of post-stroke depression with or without vitamin D deficiency

**Figure 2:**
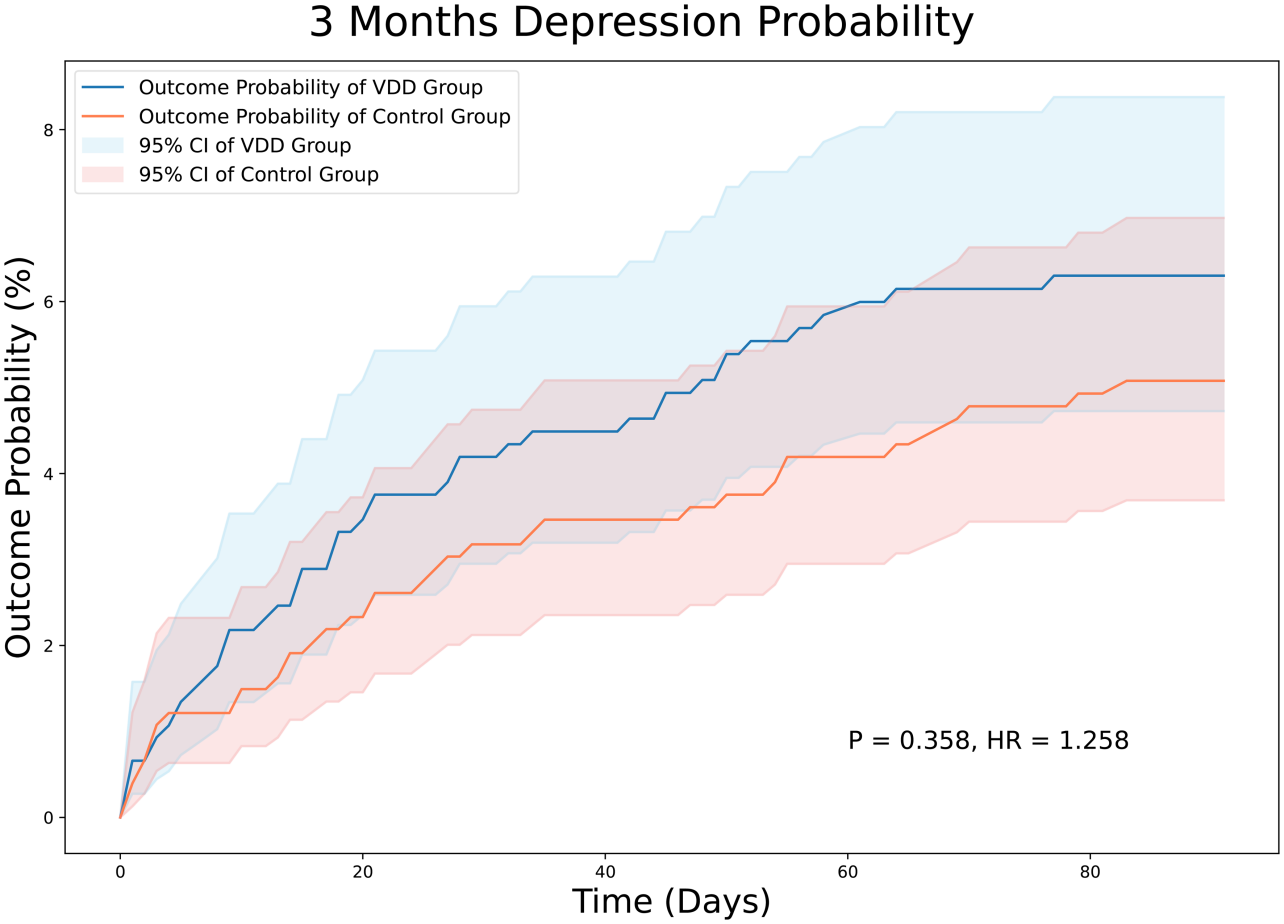
Kaplan–Meier analysis showing an estimate of the cumulative incidence of post-stroke depression among patients with vitamin D deficiency after 3 month follow-up. VDD, vitamin d deficiency.

**Figure 3:**
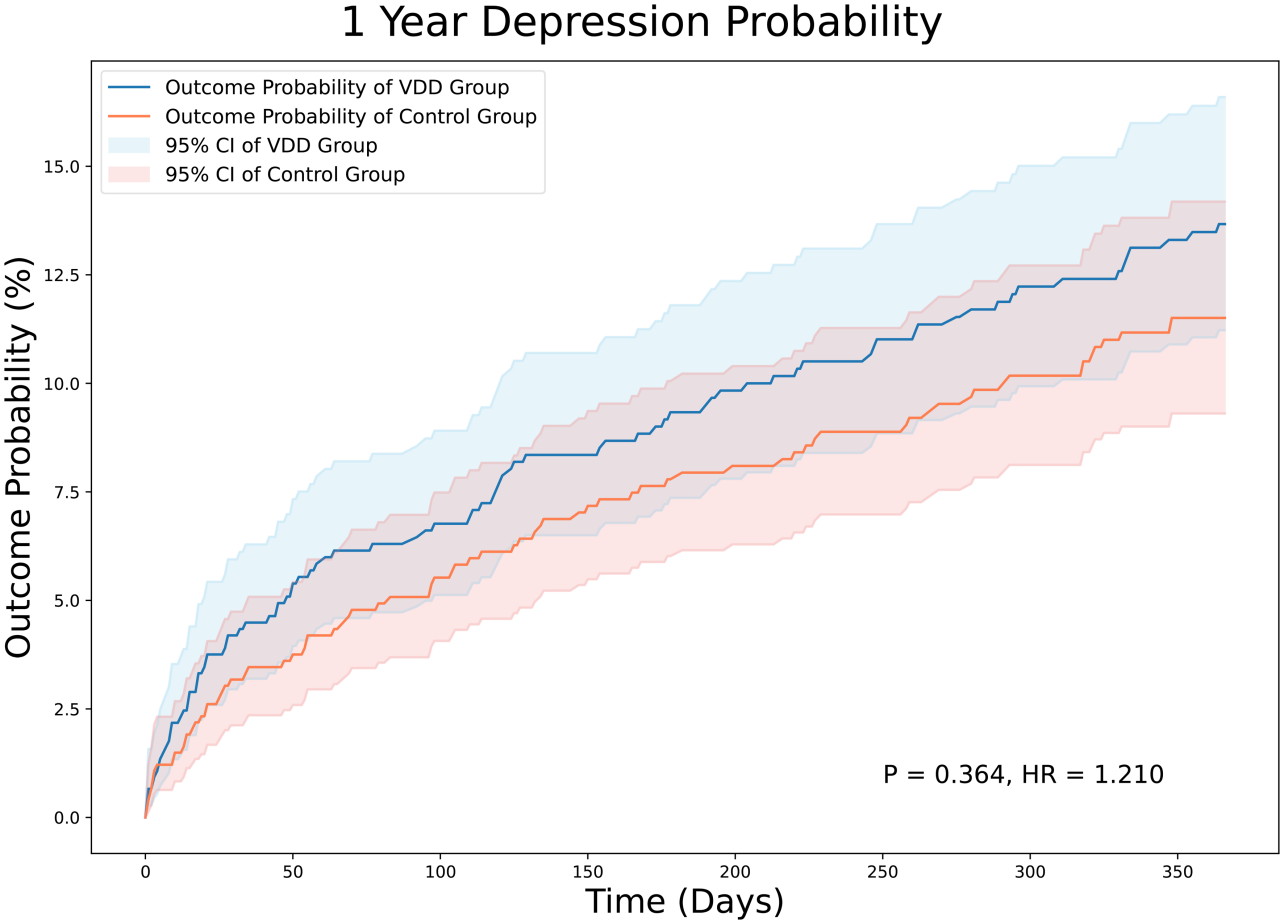
Kaplan–Meier analysis showing an estimate of the cumulative incidence of post-stroke depression among patients with vitamin D deficiency after 12 month follow-up. VDD, vitamin d deficiency.

At the 3-month follow-up, there were 44 and 36 patients in the VDD and control groups, respectively, with PSD. The incidence of PSD at the 3-month follow-up was not significantly different between the two groups (5.8% vs. 4.7%, *p* = 0.358). The Kaplan–Meier curves of survival probability also showed no significant difference in the risk of PSD (HR = 1.258; 95% CI, 0.810–1.954, *p* = 0.358) (Figure 2). Similarly, the 12-month follow-up did not reveal statistically significant differences between the two groups (11.6% vs. 10.2%, *p* = 0.364). Consistently, Kaplan–Meier curves indicated no difference between the VDD and control groups. (HR = 1.210; 95% CI, 0.891–1.642, *p* = 0.364) (Figure 3).

The sensitivity analysis, which excluded stroke survivors who used psychoactive substances, yielded consistent findings during the 12-month follow-up period (HR = 0.745; 95% CI, 0.467–1.191, *p* = 0.217). In addition, sensitivity analysis of stroke subtype (i.e., hemorrhagic stroke vs. ischemic stroke) showed that VDD was not associated with an increased risk of PSD in patients with hemorrhagic stroke (HR = 0.956; 95% CI, 0.369–2.478, *p* = 0.926) or ischemic stroke (HR = 0.712; 95% CI, 0.291–1.742, *p* = 0.455) at the 12-month follow-up.

### 3.4. Cumulative risk of pneumonia and emergency department visit with or without vitamin D deficiency

**Figure 4:**
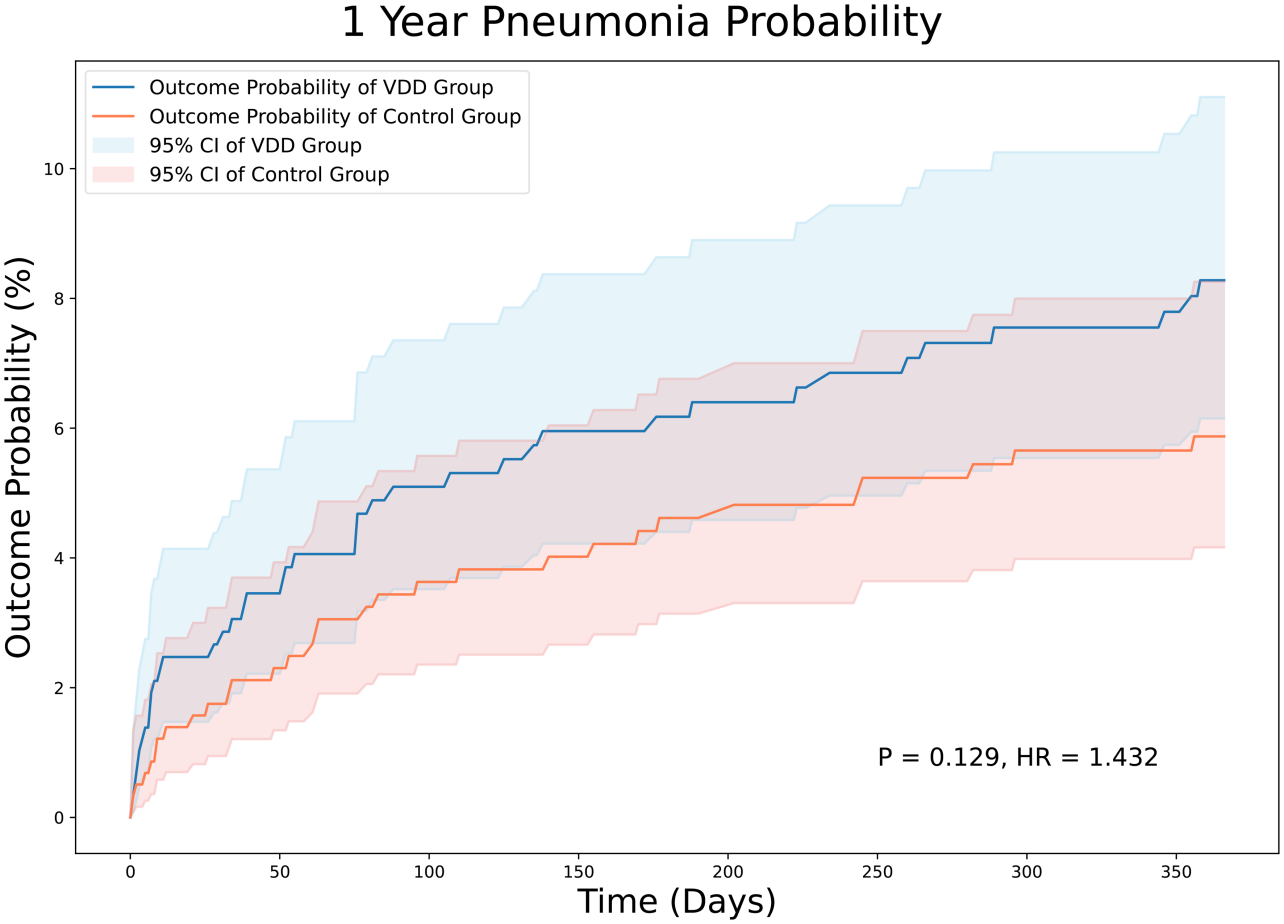
Kaplan–Meier analysis showing an estimate of the cumulative incidence of pneumonia among patients with vitamin D deficiency after 12 month follow-up. VDD, vitamin d deficiency.

At the 12-month follow-up, stroke survivors with VDD had a similar risk of pneumonia (HR = 1.053; 95% CI, 0.671–1.65, *p* = 0.823) (Figure 4) and emergency department visits (HR = 1.206; 95% CI, 0.935–1.554, *p* = 0.148) (data not shown) compared to those without VDD.

## 4. Discussion

PSD is a multifactorial disease involving several non-modifiable (e.g., genetic) and modifiable (e.g., environmental) risk factors (1). The early identification of modifiable risk factors is critical for the prevention and treatment of PSD. In contrast to previous observational studies (2, 3, 30), the current study is the first to address this issue based on pooled data predominantly from a Western population. Following PSM to adjust for confounding factors, the present study identified 758 patients each in the VDD and control groups from a base population of 1,865,486 patients with stroke. Our results showed no significant differences in the incidence of PSD between the two groups at the 3-month and 12-month follow-up. Furthermore, VDD was not related to an increased risk of pneumonia or emergency department visits at the 12-month follow-up. The three leading comorbidities in both groups were musculoskeletal disorders, metabolic diseases, and hypertension. Overall, our study found no association between VDD and PSD risk.

Previous studies have suggested that PSD is a multifactorial disease involving genetic and environmental factors (33). For instance, genetic factors such as serotonin transporter gene (SERT) polymorphisms have been linked to PSD (1, 34). In addition, age remains a debatable risk factor because of the lack of robust evidence to support its association with PSD (35). In contrast to non-modifiable factors, an understanding of the modifiable risk factors for PSD is crucial for its prevention and treatment, as well as for unraveling the disease mechanism. In addition to the identification of stroke severity, pre-stroke depression, and cognitive impairment following stroke, some consistent risk factors for PSD, medical comorbidities, and adverse psychosocial factors, such as weak social support and defective coping strategies, have been shown to be associated with an increased risk. A previous meta-analysis that included 36 observational studies identified a history of mental illness as the most significant predictor of PSD, followed by age < 70 years, female sex, neuroticism, more severe stroke, presence of a family history, and a higher handicap level, while good social support was a protector against the development of PSD (36). A recent observational study enrolling 5,882 patients also revealed an increased likelihood of PSD development in women, those with a history of previous stroke or myocardial infarction, and those with Medicaid insurance (37).

Several studies have addressed the potential role of vitamin D supplements in reducing the risk of depression (38, 39). For example, in a study involving 78 older adults (aged over 60 years) with moderate to severe depression, participants were randomly assigned to receive either weekly vitamin D3 supplementation (50,000 U) for 8 weeks or a placebo. The results indicated that vitamin D supplementation led to improved depression scores in this population, suggesting its potential effectiveness in alleviating depressive symptoms in older adults (39). Several recent studies on the Asian population have attempted to investigate the association between vitamin D levels and the risk of PSD (2, 3, 30). A study on 442 stroke survivors reported a higher risk of PSD in patients with VDD and insufficiency than in those with normal vitamin D levels at one-month follow-up (2). Another small-scale study involving 189 individuals with acute ischemic stroke reported an independent relationship between low serum vitamin D levels (≤37.1 nmol/L) and the occurrence of PSD during one-month follow-up (3). However, another study recruiting 233 stroke patients found that pre-stroke poor sleep quality, instead of VDD, contributed to the development of PSD (40). Consistently, a study of 126 stroke survivors reported only a weak association between VDD and symptoms of depression (41). Therefore, these findings indicate an ambiguous association between VDD and PSD risk.

The current study, which is the first to investigate the link between vitamin D levels and the risk of PSD based on a nationwide database focusing on the western population, found no association between VDD and the risk of PSD. The strengths of the present study include the use of PSM to adjust for potential confounding factors to increase the validity of our results, as well as the large sample size of multiple healthcare organizations. In addition, considering the racial differences in the association between low circulating vitamin D levels and the risk of PSD (33), the predominance of Western populations in the current study instead of Asians in previous reports (2, 3, 30) allowed extrapolation of our findings to Western countries.

The incidence of PSD has increased in recent years owing to the growing number of stroke survivors (1). Based on prospective studies on the Asian population, it is estimated that approximately one-third (i.e., 26.6–29.1%) of stroke survivors suffer from PSD (2, 3). A large cohort study (4) that included 157,243 stroke patients discovered that over a quarter (25.4%) of stroke patients developed depression based on the diagnosis at hospital discharge or the use of antidepressants within 2 years after stroke. According to a meta-analysis of 61 studies involving 25,488 patients, the pooled prevalence of depression was found to be even higher at 31%, despite a wide range (5–84%) (42). Such a large discrepancy in the incidence of PSD may be attributed to variations in depression assessment criteria, timing of assessment after stroke, sample size, and inclusion and exclusion criteria in published studies. For instance, some studies in that meta-analysis did not exclude patients with aphasia, previous stroke, or a history of depression (43–45).

In the current study, the incidence of PSD was 4.7 and 10.2% in the control group at the 3-month and 12-month follow-ups, respectively. There are two possible explanations for the relatively low incidence of PSD in the current study. First, we excluded patients with a high risk of PSD, including those with a history of stroke, dementia, Parkinson’s disease, dysphasia, aphasia, osteoporosis, or major depression. Second, the possibility of an underestimation of the incidence of PSD in the Electronic Health Record database due to failure to meet the diagnostic criteria of PSD cannot be excluded. Consistently, a recent study focusing on 5,882 patients reported a relatively low cumulative incidence of PSD (i.e., 6.4%) at 1 year for the entire cohort based on data from Geisinger’s Electronic Health Record (37).

Exploring the potential therapeutic benefits of vitamin D supplementation in PSD management is an important avenue to consider. However, the impact of vitamin D supplementation on PSD improvement remains uncertain because of the lack of robust evidence. In a 12-week randomized trial with 40 patients undergoing intensive neurorehabilitation after stroke, those receiving 2000 IU/day of oral cholecalciferol showed no significant additional benefit in mood (e.g., depression) and functional recovery compared with the control group without vitamin D supplementation (46). The authors suggested that the positive effects observed were mainly attributed to neurorehabilitation treatment rather than vitamin D supplementation (46). Owing to the limited number of patients in that study, further research is imperative to address this issue and gain a more comprehensive understanding of the potential benefits of vitamin D supplementation in managing PSD.

The current study has several limitations. First, the limited number of patients (i.e., 758 in both groups) precluded our elucidation of the potential association between VDD and the risk of PSD. Second, while using the ICD-10 code for identifying patients with PSD from electronic health record (EHR) data is a practical approach, the absence of detailed diagnostic assessments does indeed represent a limitation in our study. Third, we were unable to control for factors that were not included in the electronic health records, such as lifestyle factors (e.g., use of dietary supplements, smoking, and alcohol consumption), degree of social support, and level of physical activity. Fourth, the retrospective nature of the study design was subject to potential bias from uncontrolled confounders. Fifth, the use of data on vitamin D levels limited to within 3 months before stroke for diagnosing VDD could not rule out the possibility of restoration to a normal level after stroke and vice versa. Finally, it is crucial to acknowledge that the presence of a real effect or relationship cannot be completely ruled out due to the confounding factors and limitations mentioned earlier. Thus, cautious interpretation of the results and consideration of potential uncertainties are essential for drawing accurate conclusions from the research findings.

In conclusion, this study found no significant association between VDD and the risk of PSD at the 3-month and 12-month follow-ups, suggesting that VDD may not be a significant risk factor for PSD. Furthermore, our findings indicated that VDD was not associated with an increased risk of pneumonia or emergency department visits during the 12-month follow-up period. Further large-scale prospective studies are warranted to elucidate the potential protective effects of vitamin D against PSD and verify our findings.

## Data availability statement

The raw data supporting the conclusions of this article will be made available by the authors, without undue reservation.

## Ethics statement

The studies were conducted in accordance with the local legislation and institutional requirements. The current study was approved by the Institutional Review Board of the Chi Mei Medical Center (IRB Serial No. 11203–010). Only de-identified patient records were collected and no individually identifiable information was used, collected, or transmitted. Therefore, written informed consent of participants is not required for current study.

## Author contributions

C-NH, J-YW, and K-CH: conceptualization and literature search. C-KS and J-YC: methodology. C-NH, Y-JC, and I-WC: data analysis. I-WC and C-KS: data extraction. K-CH and C-KS: writing—original draft preparation. I-WC, C-KS, and K-CH: writing—review and editing. All the authors have read and agreed to the published version of the manuscript.

## Funding

This research was funded by the Chi Mei Medical Center, Tainan, Taiwan (grant number: CMOR11203). APC was funded by CMOR 11203.

## Conflict of interest

The authors declare that the research was conducted in the absence of any commercial or financial relationships that could be construed as a potential conflict of interest.

## Publisher’s note

All claims expressed in this article are solely those of the authors and do not necessarily represent those of their affiliated organizations, or those of the publisher, the editors and the reviewers. Any product that may be evaluated in this article, or claim that may be made by its manufacturer, is not guaranteed or endorsed by the publisher.
